# Comparison between ultrasound and electromyography for detection of fasciculations in amyotrophic lateral sclerosis: an updated systematic review and meta-analysis

**DOI:** 10.1590/0100-3984.2026.0011

**Published:** 2026-09-25

**Authors:** Márcio Luís Duarte, Wagner Iared, Acary Bulle Souza Oliveira, Lucas Ribeiro dos Santos, Maria Stella Peccin da Silva

**Affiliations:** 1 Department of Radiology, Universidade de Ribeirão Preto - Campus Guarujá, Guarujá, SP, Brazil; 2 Department of Radiology, Diagnósticos da América S/A, São Paulo, SP, Brazil; 3 Instituto de Ensino e Pesquisa DASA (IEPD), São Paulo, SP, Brazil; 4 Department of Ultrasonography, Diagnósticos da América S/A, São Paulo, SP, Brazil; 5 Department of Neurology, Universidade Federal de São Paulo/Escola Paulista de Medicina (UNIFESP/EPM), São Paulo, SP, Brazil; 6 Department of Internal Medicine, Universidade de Ribeirão Preto - Campus Guarujá, Guarujá, SP, Brazil; 7 Department of Human Movement Sciences and Postgraduate Program in Evidence-Based Health, Universidade Federal de São Paulo (UNIFESP), São Paulo, SP, Brazil

**Keywords:** Ultrasonography, Electromyography, Fasciculation, Amyotrophic lateral sclerosis, Motor neuron disease., Ultrassonografia, Eletromiografia, Fasciculação, Esclerose lateral amiotrófica, Doenças do neurônio motor.

## Abstract

Fasciculations are a key manifestation of lower motor neuron dysfunction in amyotrophic lateral sclerosis (ALS). Al-though electromyography is considered the gold-standard method for the detection of fasciculations, its sensitivity for that purpose depends on the muscle sampled and the examination time. Muscle ultrasound has emerged as a dynamic, noninvasive technique with high sensitivity for detecting spontaneous muscle activity. This updated system-atic review compares ultrasound and electromyography in terms of their performance for the detection of muscle fas-ciculations in ALS, with stratification by muscle group and observation time. A comprehensive search of the literature identified studies evaluating both techniques. Data were synthesized descriptively and, when possible, quantitatively. Ultrasound demonstrated higher or comparable detection rates across most muscle groups, particularly in muscles that are large or deep, such as the biceps brachii, tibialis anterior, and vastus lateralis. Longer ultrasound observation times, especially 60 s, were associated with substantially higher detection rates without a loss of specificity. Although electromyography is still essential for electrophysiological characterization, ultrasound offers broader muscle cover-age, excellent reproducibility, and a noninvasive approach. These findings support muscle ultrasound as a valuable complementary tool for the detection and assessment of fasciculations in patients with motor neuron disease.

## Abstract (pt)

As fasciculações representam uma manifestação central da disfunção do neurônio motor inferior na esclerose lateral amiotrófica (ELA). A eletroneuromiografia é considerada o método de referência para sua detecção, porém sua sensibi-lidade depende da extensão da amostragem muscular e do tempo de exame. A ultrassonografia muscular surgiu como uma técnica dinâmica e não invasiva, com elevada sensibilidade para identificar atividade muscular espontânea. Esta revisão sistemática atualizada compara o desempenho da ultrassonografia e da eletroneuromiografia na detecção de fasciculações em pacientes com ELA, com estratificação por grupos musculares e tempo de observação. A literatura disponível foi analisada de forma descritiva e, quando possível, quantitativa. De modo geral, a ultrassonografia apre-sentou taxas de detecção superiores ou semelhantes às da eletroneuromiografia na maioria dos músculos, especial-mente em músculos grandes ou profundos, como bíceps braquial, tibial anterior e vasto lateral. Protocolos com maior tempo de observação, particularmente 60 s, aumentaram significativamente a taxa de detecção pela ultrassonografia sem comprometer a especificidade. Embora a eletroneuromiografia permaneça fundamental para a caracterização ele-trofisiológica, a ultrassonografia oferece maior cobertura muscular, excelente reprodutibilidade e caráter não invasivo, configurando-se como método complementar valioso na avaliação das doenças do neurônio motor.

## INTRODUCTION

Fasciculations are brief, spontaneous twitches of muscle fibers innervated by a single motor unit^**(^1^)**^. They may occur in a wide spectrum of conditions, including benign conditions-such as benign fasciculation syn-drome, physiological variants, metabolic disturbances, and drug-induced states-as well as in pathological conditions requiring accurate diagnosis, including amyo-trophic lateral sclerosis (ALS), other motor neuron dis-eases, spinal cord lesions, and neuromyotonia. Fascicu-lations are not a typical feature of primary myopathies, given that most originate from dysfunction of the anterior horn cell or distal motor nerve terminal^**(^2^)**^. Fascicula-tions can be identified clinically, electrophysiologically (by electromyography), or visually (by ultrasonography). Each method offers complementary insights into lower motor neuron (LMN) dysfunction^**(^3^)**^.

Fasciculations arise from spontaneous depolariza-tion of unstable LMN or terminal axons, generating involuntary discharges that activate a single motor unit. Although magnetic resonance imaging techniques, in-cluding advanced motor unit assessment and diffu-sion-weighted imaging, have also been explored for detecting neuromuscular abnormalities associated with fasciculations, they still play a complementary role and are not always accessible in routine clinical practice. The discharges that provoke fasciculations may result from membrane hyperexcitability, collateral sprouting, ion-channel dysfunction, or impaired axonal integrity-mechanisms commonly associated with denervation and reinnervation processes^**(^2^)**^. In ALS, fasciculations reflect ongoing motor-unit instability and increased axonal excitability, often preceding clinically detectable weakness and therefore representing one of the earliest indicators of LMN degeneration^**(^3^)**^.

Clinically, fasciculations carry substantial diagnos-tic relevance in ALS. Their presence in multiple body regions, especially when associated with weakness, at-rophy, and upper motor neuron signs, increases the likelihood of ALS and contributes to meeting the Awaji and revised El Escorial criteria^**(^4^-^6^)**^. Fasciculations in specific muscle groups-such as the tongue, paravertebral, and distal limb muscles-are particularly suggestive of motor neuron disease. Early and accurate detection of fasciculations can therefore shorten the time to di-agnosis, guide disease monitoring, and facilitate the differentiation from benign fasciculation syndromes and other neuromuscular disorders.^**(^2^,^3^,^7^)**^.

Electromyography is still the gold-standard test for evaluating LMN impairment in ALS^**(^8^)**^. However, it has inherent limitations-its sensitivity depends on the number of muscles examined and the duration of screen-ing for each muscle^**(^9^)**^. In addition, electromyography primarily samples small areas of muscle and cannot assess atrophy or structural integrity^**(^10^)**^. Ultrasound, in contrast, is a dynamic, noninvasive, and highly motion-sensitive technique capable of detecting displacements as small as 5 µm, with frame rates exceeding 80 fps^**(^11^)**^. It allows real-time visualization of superficial and deep muscles, enabling analysis of muscle atrophy and archi-tecture, as well as fasciculations^**(^11^,^12^)**^.

A prior systematic review by our group demonstrated that ultrasound shows greater overall accuracy than electromyography for detecting fasciculations in ALS^**(^13^)**^. However, that analysis did not stratify results by muscle group or duration of the observation. Since then, ad-ditional studies have explored muscle-specific detection rates, including protocols with 60-s observations^**(^14^,^15^)**^. Given the anatomical and physiological variability in fasciculation frequency across different muscles, under-standing how electromyography and ultrasound perform within specific muscle groups may guide the standardiza-tion of imaging protocols and optimize diagnostic yield. Therefore, the aim of this updated systematic review and meta-analysis was to compare electromyography and ultrasound in terms of the rate of detection of fascicula-tions across individual muscles, integrating all available evidence up to December 24, 2025.

## METHODS

### Study design

This was a systematic review of diagnostic perfor-mance studies comparing ultrasound and electromyog-raphy for the detection of muscle fasciculations in ALS.

This review represents an updated synthesis expand-ing on our previously published systematic review^**(^12^)**^, incorporating newly available evidence and extended observation protocols.

The review was conducted in accordance with the Preferred Reporting Items for Systematic Reviews and Meta-Analyses guidelines^**(^16^)**^, registered on the Inter-national Prospective Register of Systematic Reviews platform (Registration no. CRD42017078388), and approved by the local institutional research ethics com-mittee. Because the study involved no human or animal subjects, the institutional review board waived the re-quirement for formal institutional review.

### Eligibility criteria

We included studies that directly compared ultra-sound and electromyography as means of detecting fasciculations in patients with ALS, reporting absolute numbers of muscles or patients with and without fas-ciculations for each method. All study designs (prospec-tive, cross-sectional, and case-control) were eligible if muscle-specific data were available. No restrictions were applied regarding language or publication status.

### Study populations

Studies enrolling adults diagnosed with ALS or its variants, according to the Awaji or revised El Es-corial criteria, were included. Studies limiting their samples to healthy volunteers or patients with other neuromuscular conditions were excluded from quan-titative synthesis but could be discussed qualitatively for methodological comparison.

### Index and comparator tests

####  Ultrasound 

On ultrasound, fasciculations were defined as spon-taneous, irregular, brief contractions of muscle fibers observed in real time on B-mode imaging. Observation times of 10, 30, or 60 s per muscle were accepted.

####  Electromyography 

On electromyography, fasciculations were defined as spontaneous discharges of motor unit potentials in relaxed muscle.

### Reference standard

Because all participants had a confirmed diagnosis of ALS, the disease served as the clinical context but not as the diagnostic reference standard. The objective was to compare electromyography and ultrasound in terms of their performance in the detection of fasciculations, not their diagnostic accuracy for ALS itself.

### Search strategy

A comprehensive search was conducted across the MEDLINE (via PubMed), EMBASE, Cochrane Library, and Latin-American and Caribbean Health Sciences Literature databases, from their inception to December 24, 2025. The search combined controlled vocabulary and free-text terms for “fasciculation”, “ultrasonogra-phy”, and “electromyography” (Supplemental material). Manual searches of the bibliographies of all included articles and relevant reviews were conducted to identify additional studies.

**SUPPLEMENTAL MATERIAL:** 

Database	Search strategy
**Cochrane library**	#1: MeSH descriptor: [Fasciculation] explode all trees #2: MeSH descriptor: [Ultrasonography] explode all trees #3: MeSH descriptor: [Electromyography] explode all trees #4: #1 AND #2 AND #3
**Pubmed / Medline**	#1: (“Fasciculation” [MeSH]) OR (Fasciculations) OR (Fasciculation, Neural) OR (Fasciculations, Neural) OR (Neural Fasciculation) OR (Neural Fasciculations) OR (Fasciculation, Muscular) OR (Fasciculations, Muscular) OR (Muscular Fasciculation) OR (Muscular Fasciculations) OR (Fasciculation, Skeletal Muscle) OR (Fasciculations, Skeletal Muscle) OR (Muscle Fasciculation, Skeletal) OR (Muscle Fasciculations, Skeletal) OR (Skeletal Muscle Fasciculation) OR (Skeletal Muscle Fasciculations) OR (Fasciculation, Tongue) OR (Fasciculations, Tongue) OR (Tongue Fasciculation) OR (Tongue Fasciculations) OR (Fasciculation, Benign) OR (Benign Fasciculation) OR (Benign Fasciculations) OR (Fasciculations, Benign) #2: (“Ultrasonography” [MeSH]) OR (Echotomography) OR (Diagnostic Ultrasound) OR (Diagnostic Ultrasounds) OR (Ultrasound, Diagnostic) OR (Ultrasounds, Diagnostic) OR (Sonography, Medical) OR (Medical Sonography) OR (Ultrasound Imaging) OR (Imaging, Ultrasound) OR (Imagings, Ultrasound) OR (Ultrasound Imagings) OR (Echography) OR (Ultrasonic Imaging) OR (Imaging, Ultrasonic) OR (Echotomography, Computer) OR (Computer Echotomography) OR (Tomography, Ultrasonic) OR (Ultrasonic Tomography) OR (Diagnosis, Ultrasonic) OR (Diagnoses, Ultrasonic) OR (Ultrasonic Diagnoses) OR (Ultrasonic Diagnosis) #3: (“Electromyography” [MeSH]) OR (Electromyographies) OR (Surface Electromyography) OR (Electromyographies, Surface) OR (Electromyography, Surface) OR (Surface Electromyographies) #4: #1 AND #2 AND #3
**EMBASE**	#1: ‘fasciculation’/exp OR ‘muscle fasciculation’ #2:’electromyography’/exp OR ‘electric myography’ OR ‘electrical myography’ OR ‘electro myography’ OR ‘myography, electric’ OR ‘quantitative electromyography’ OR ‘surface electromyography’ #3:’echography’/exp OR ‘doptone’ OR ‘duplex echography’ OR ‘echogram’ OR ‘echoscopy’ OR ‘echosound’ OR ‘high resolution echography’ OR ‘scanning, ultrasonic’ OR ‘sonogram’ OR ‘sonography’ OR ‘ultrasonic detection’ OR ‘ultrasonic diagnosis’ OR ‘ultrasonic echo’ OR ‘ultrasonic examination’ OR ‘ultrasonic scanning’ OR ‘ultrasonic scintillation’ OR ‘ultrasonography’ OR ‘ultrasound diagnosis’ OR ‘ultrasound scanning’ #4: #1 AND #2 AND #3
**LILACS**	#1: mh:“Fasciculação” OR (Fasciculación) OR (Fasciculation) OR (Fasciculações) OR (mh:C10.597.613.250$) OR (mh:C23.888.592.608.250$) #2: mh:“Ultrassonografia” OR (Ultrasonografía) OR (Ultrasonography) OR (Échographie) OR (Ecografia) OR (Ecotomografia Computador) OR (Sonografia Médica) OR (Ecografia Médica) OR (Tomografia Ultrassônica) OR (Diagnóstico Ultrassom) OR (Imagem Ultrassônica) OR (Imagem Ultrassonográfica) OR (Imagem Ultrassom) OR (Imagem Ultrassom) OR (Ecotomografia) OR (mh:E01.370.350.850$) #3: mh:“Eletromiografia” OR (Electromiografía) OR (Electromyography) OR (Électromyographie) OR (mh:E01.370.405.255$) OR (mh:E01.370.530.255$) #4: #1 AND #2 AND #3

### Study selection and data extraction

Two reviewers, working independently, screened all titles and abstracts, retrieved full-text articles, and extracted data using a standardized form. Discrepancies were resolved by consensus with a third reviewer. The selection process was carried out on the Rayyan platform (https://rayyan.qcri.org), as previously described^**(^17^)**^. The following data were extracted: publication year, authors, and country; study design and sample size; muscles examined and observation time per muscle; and the numbers of positive and negative fasciculation detections for each method (ultrasound and electromyography).

### Quality assessment

Although all included studies evaluated patients with clinically confirmed ALS, the main objective of this update was not to determine diagnostic accuracy for the disease itself, but rather to compare the techni-cal performance of ultrasound and electromyography in detecting muscle fasciculations. Therefore, no clinical gold standard was applied for diagnostic validation, and the comparison focused solely on the relative difference in detection performance between the two techniques. In this context, the Quality Assessment of Diagnostic Accuracy Studies 2 tool-designed for assessing bias in diagnostic accuracy studies-was deemed inappropri-ate for the scope of this review. Instead, we employed only the Research Triangle Institute item bank, because it is more suitable for observational and comparative performance studies, allowing a more precise evalua-tion of methodological clarity, population description, and analytical consistency of the included works^**(^18^,^19^)**^.

### Statistical analysis

For each study and muscle group, the fascicula-tion detection rate was calculated as the proportion of muscles showing visible or recordable fasciculation events relative to the total number of muscles examined. Comparisons between ultrasound and electromyography were expressed as absolute numbers and percentages.

When at least two studies evaluated the same muscle, pooled detection rates for each method were calculated by using RevMan 5.3 (Cochrane Collaboration, Oxford, UK) and Meta-DiSc 1.4 (Hospital Ramón y Cajal, Madrid, Spain). If only one study provided data for a given muscle, the results are presented descriptively. Comparisons were stratified by muscle group (e.g., tongue, biceps brachii, and tibialis anterior) and the duration of observation (10, 30, and 60 s). Risk ratios with 95% confidence intervals were used as the effect measure. For homogeneous sub-groups (e.g., analyses restricted to a single observation time or muscle), pooled estimates were calculated with the Mantel-Haenszel fixed-effects model. When het-erogeneity across studies or muscles was expected (e.g., when pooling all muscles within the same observation time), the Mantel-Haenszel random-effects model was applied. Between-study heterogeneity was assessed by calculating the I^^2^^ statistic, with values above 40% being considered indicative of moderate-to-high heterogeneity.

## RESULTS

The initial search retrieved 176 records related to the topic in question (Figure 1). After titles, abstracts, and full texts had been screened, nine studies met all of the inclusion criteria and were included in the final analysis. Three studies, despite having compared elec-tromyography and ultrasound, were excluded: a study conducted by Regensburger et al.^**(^20^)**^ was excluded be-cause ultrasound and electromyography were performed simultaneously by the same operator, thus precluding adequate blinding between methods and introducing potential observer bias; Botter et al.^**(^21^)**^ was excluded because it was a technical study conducted exclusively in healthy volunteers rather than ALS patients; and a study conducted by Bibbings et al.^**(^22^)**^, developed by the Sheffield group, was excluded because it compared ultrasound and electromyography using an automated video-analysis system instead of a human observer, thus allowing assessment of temporal effects and methodological innovations not previously addressed. Compared with our previous systematic review^**(^13^)**^, this update included two additional eligible studies-both extending the ultrasound observation time to 60 s^**(^14^,^15^)**^.

**Figure 1:**
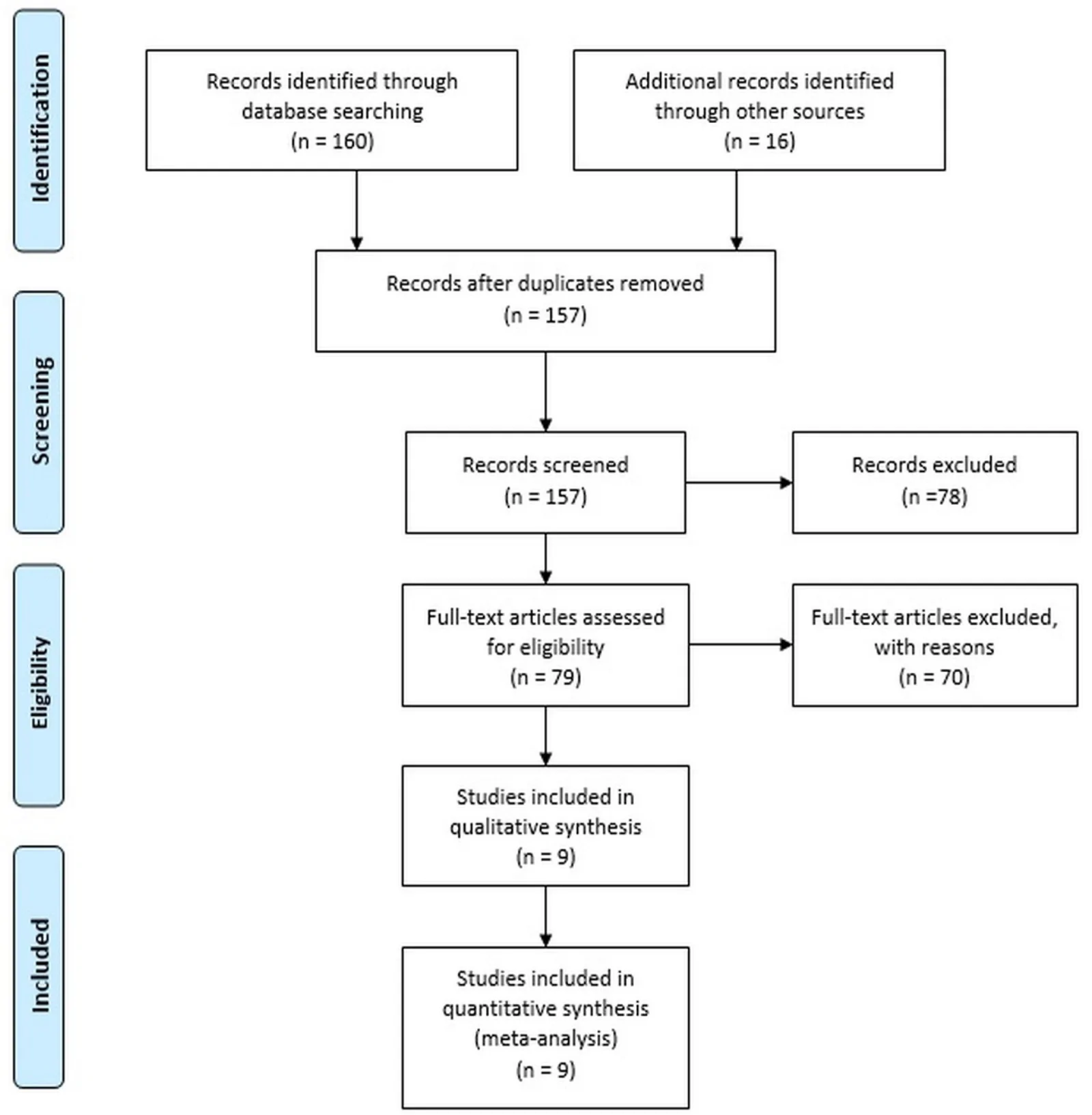
Preferred Reporting Items for Systematic Reviews and Meta-Analyses flow chart.

### Evaluation with electromyography and 10-s ul-trasound, by muscle

Two studies-Arts et al.^**(^23^)**^ and O’Gorman et al.^**(^24^)**^-evaluated sternocleidomastoid muscles (N = 135, collectively) and reported exact numbers of the muscles testing positive for fasciculations. In those two studies (collectively), fasciculations were detected in four (12.1%) of 33 muscles evaluated by electro-myography and in 27 (26.4%) of 102 muscles evalu-ated for 10 s on ultrasound (Figure 2A). The tongue was also evaluated in two studies-Grimm et al.^**(^10^)**^ and O’Gorman et al.^**(^24^)**^-collectively comprising 215 muscles, also evaluated. In those two studies, fas-ciculations were found in 47 (55.4%) of 85 muscles evaluated by electromyography and in 88 (67.7%) of 130 muscles evaluated by 10-s ultrasound (Figure 2B). When all muscles were evaluated together-in Grimm et al.^**(^10^)**^, Johansson et al.^**(^25^)**^, O’Gorman et al.^**(^24^)**^, and Reimers et al.^**(^9^)**^; N = 1,957)-fascicula-tions were identified in 339 (48.8%) of 695 muscles evaluated by electromyography and in 627 (49.7%) of 1,262 muscles evaluated by 10-s ultrasound, showing comparable detection rates between the two modali-ties (Figure 2C).

**Figure 2:**
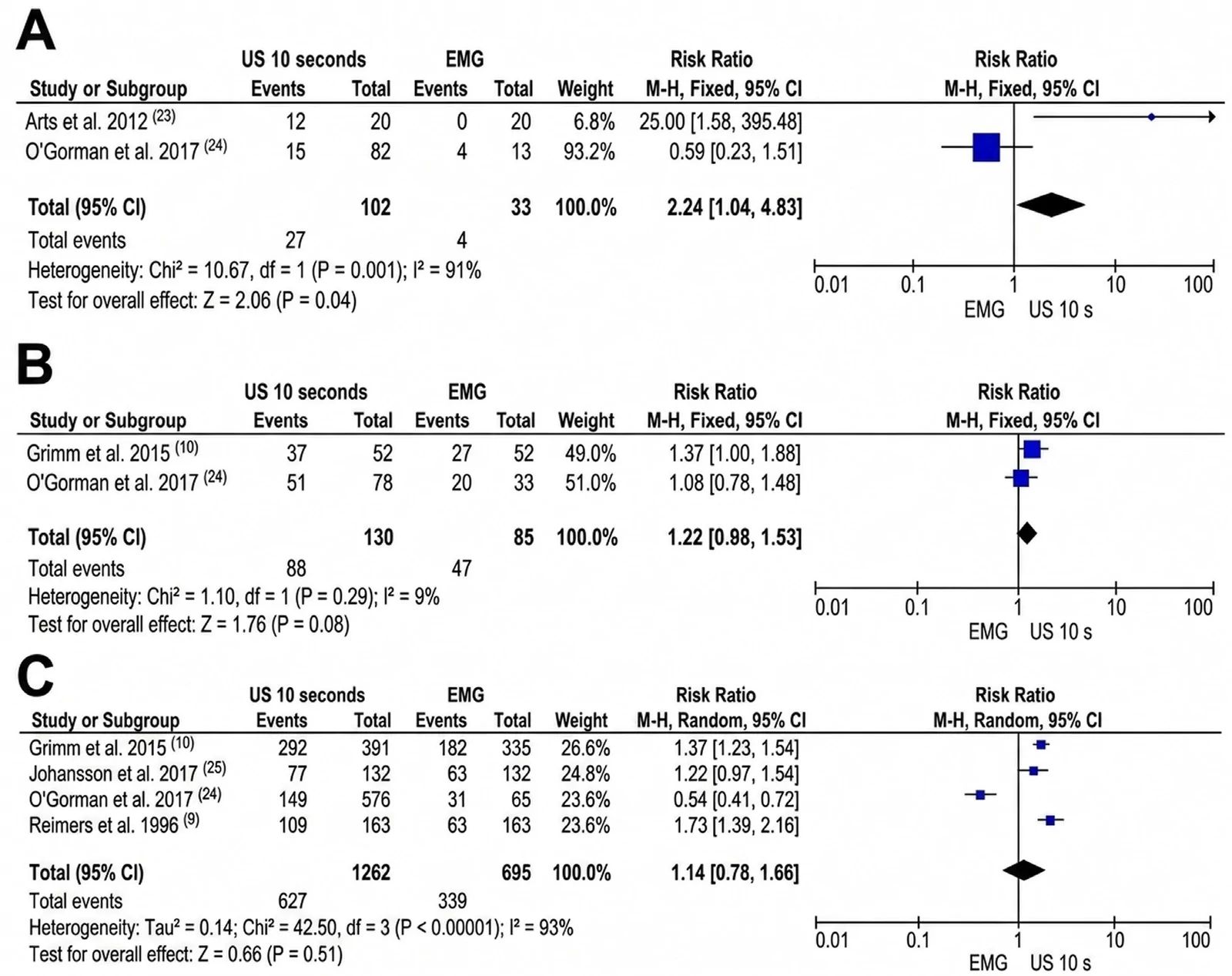
Comparison between ultrasound (US, 10-s scan) and electromyography (EMG) for detection of fasciculations in the sternoclei-domastoid muscle (**A**), the tongue (**B**), and all muscles combined (**C**).

M-H, Mantel-Haenszel; df, degrees of freedom.

### Evaluation with electromyography and 30-s ul-trasound, by muscle

Three studies-Misawa et al.^**(^26^)**^, Zamani et al.^**(^27^)**^, and O’Gorman et al.^**(^24^)**^- provided data on muscle-specific comparisons between electromyography and 30-s ultrasound. For the tongue, fasciculations were detected in 36 (11.2%) of 153 muscles evaluated by electromyography and in 106 (63.1%) of 168 muscles evaluated by 30-s ultrasound. Misawa et al.^**(^26^)**^ re-ported that, in 81 patients, electromyography identified no fasciculations, because of continuous voluntary contraction or respiratory activity (Figure 3A). For the biceps brachii, fasciculations were detected in 73 (60.8%) of 120 muscles evaluated by electromyography and in 105 (87.5%) of 120 muscles evaluated by 30-s ultrasound (Figure 3B). For the first dorsal interos-seous, 69 (57.5%) of 120 muscles were positive for fasciculations on electromyography and 94 (78.3%) of 120 muscles were positive for fasciculations on 30-s ultrasound (Figure 3C). For the vastus lateralis, positivity for fasciculation was seen in 54 (45.0%) of 120 muscles on electromyography and in 85 (70.8%) of 120 muscles on 30-s ultrasound (Figure 3D). For the tibialis anterior, 56 (46.7%) of 120 muscles were positive on electromyography and 98 (81.7%) of 120 muscles were positive on 30-s ultrasound (Figure 3E). In the studies that considered all muscles together-Misawa et al.^**(^26^)**^, Zamani et al.^**(^27^)**^, and O’Gorman et al.^**(^24^)**^; N = 2,038-fasciculations were observed in 352 (43.3%) of 812 muscles by electromyography and in 718 (58.6%) of 1,226 muscles by 30-s ultrasound, showing a consistent increase in detection with ultrasound as the observation time doubled (Figure 3F).

**Figure 3:**
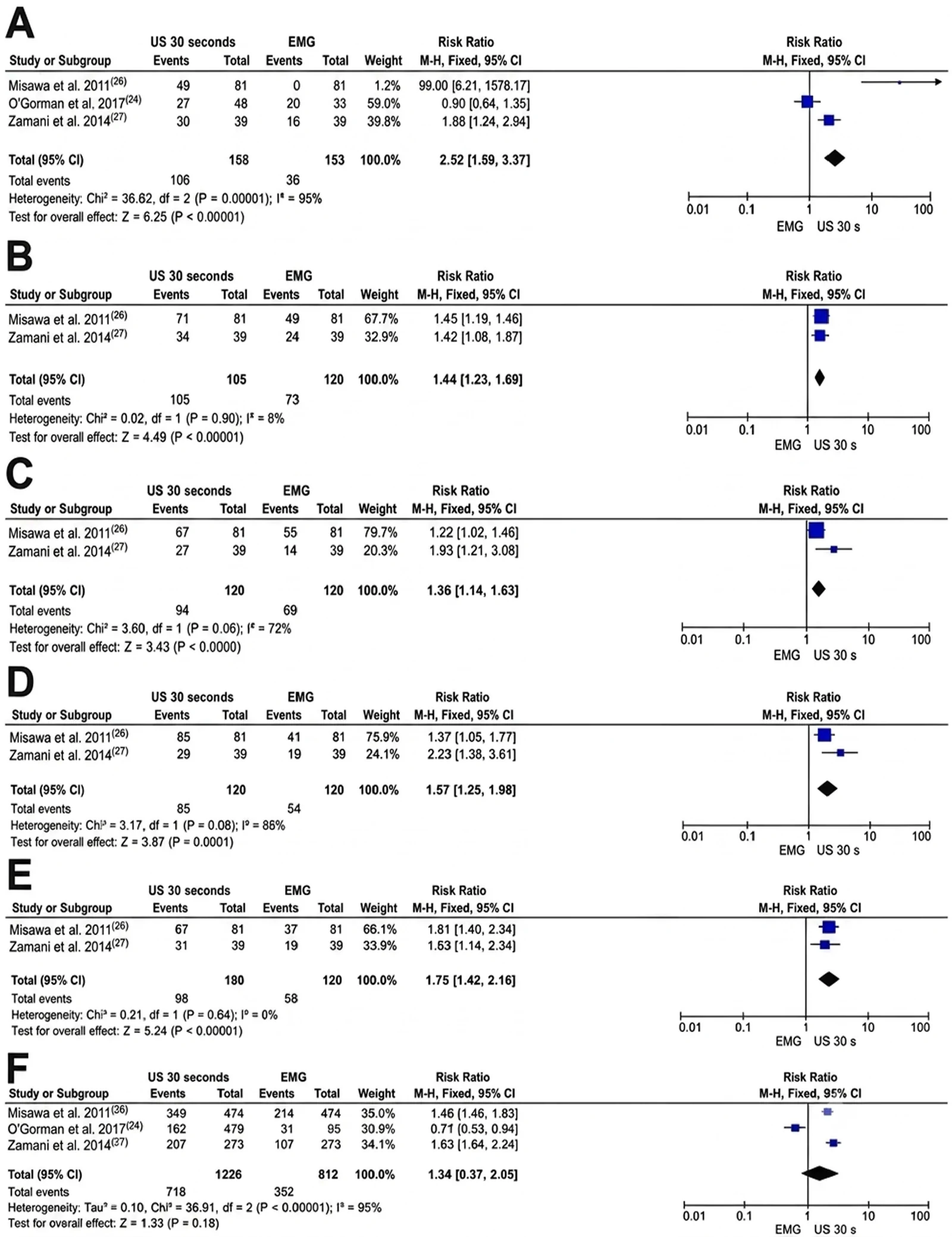
Comparison between ultrasound (US, 30-s scan) and electromyography (EMG) for detection of fasciculations in the tongue (**A**), biceps brachii (**B**), first dorsal interosseous muscle (**C**), vastus lateralis (**D**), tibialis anterior (**E**), and all muscles combined (**F**).

M-H, Mantel-Haenszel; df, degrees of freedom.

### Evaluation with electromyography and 60-s ul-trasound, by muscle

The most recent studies-Bokuda et al.^**(^14^)**^ and Ra-jula et al.^**(^15^)**^-extended the observation time to 60 s per muscle, allowing a detailed evaluation of the temporal influence on detection rates. Both studies included patients with confirmed ALS and compared electro-myography and ultrasound under blinded conditions.

####  Biceps brachii 

The biceps brachii was the only muscle assessed by both studies^**(^14^,^15^)**^, totaling 64 muscles (31 and 33, respectively).

Bokuda et al.^**(^14^)**^: fasciculations detected by 60-s ultrasound and by electromyography in 27 (87.1%) of the 31 muscles evaluated.

Rajula et al.^**(^15^)**^: fasciculations detected by 60-s ultrasound and by electromyography in 26 (78.8%) of the 33 muscles evaluated.

The combined results indicate that the detection rate for the two methods is high and equivalent when a full 60-s scan is applied in ultrasound (Figure 4).

**Figure 4:**
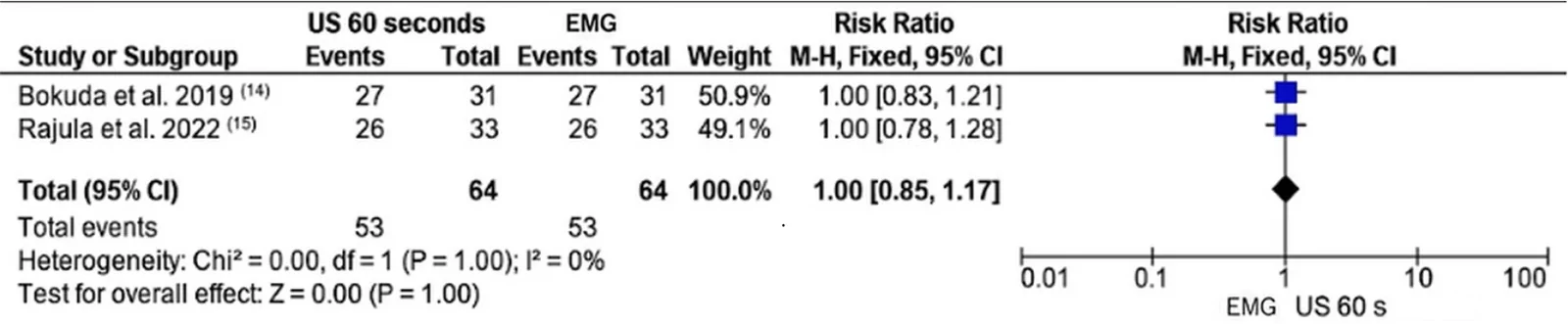
Comparison between ultrasound (US, 60-s scan) and electromyography (EMG) for detection of fasciculations in the biceps brachii.

M-H, Mantel-Haenszel; df, degrees of freedom.

####  Other muscles 

In Bokuda et al.^**(^14^)**^, seven muscles were examined: the trapezius, biceps brachii, extensor carpi radialis brevis, first dorsal interosseous, vastus medialis, tibi-alis anterior, and thoracic paravertebral muscles. Ul-trasound demonstrated higher detection rates than did electromyography in most muscles, particularly in those that are larger or deeper (Table 1).

**Table 1:** Muscles analyzed by Bokuda et al.^(^14^)^ in patients with ALS (N = 31).

	Positive for fasciculations	
	US	EMG
Muscle	n (%)	n (%)
Trapezius	25 (80.6)	24 (77.4)
Extensor carpi radialis brevis	24 (77.4)	25 (80.6)
First dorsal interosseous	27 (87.1)	26 (83.9)
Vastus medialis	26 (83.9)	23 (74.2)
Tibialis anterior	29 (93.5)	24 (77.4)
Thoracic paravertebral	31 (100.0)	24 (77.4)

EMG, electromyography; US, ultrasonography.

In Rajula et al.^**(^15^)**^, nine muscles were examined by electromyography and by 60-s ultrasound: the tongue, masseter, trapezius, biceps brachii, abductor pollicis brevis, first dorsal interosseous, vastus lateralis, tibialis anterior, and medial gastrocnemius muscles. In that study, 60-s ultrasound showed consistently higher detection rates than did electromyography in all of the muscles evaluated (Table 2). The differences in favor of ultrasound were greatest in the vastus lateralis, tibialis anterior, and gastrocnemius muscles, supporting the hypothesis that muscles that are larger or deeper require longer ultrasound scan durations for optimal detection of fasciculations.

**Table 2:** Muscles analyzed by Rajula et al.^(^15^)^ in patients with ALS (N = 33).

	Positive for fasciculations	
	US	EMG
Muscle	n (%)	n (%)
Tongue	28 (84.8)	26 (78.8)
Masseter	24 (72.7)	21 (63.6)
Trapezius	27 (81.8)	24 (72.7)
Abductor pollicis brevis	26 (78.8)	23 (69.7)
First dorsal interosseous	27 (81.8)	23 (69.7)
Vastus lateralis	29 (87.9)	23 (69.7)
Tibialis anterior	30 (90.9)	25 (75.8)
Medial gastrocnemius	28 (84.8)	21 (63.6)

EMG, electromyography; US, ultrasonography.

#### Overall detection performance 

In the two studies that employed electromyography and 60-s ultrasound, a total of 415 muscles were exam-ined: 217 in Bokuda et al.^**(^14^)**^; and 198 in Rajula et al.^**(^15^)**^. Among those 415 muscles, fasciculations were detected by ultrasound in 332 (80.0%) and by electromyography in 270 (65.0%), translating to an absolute difference of 15 percentage points in favor of ultrasound. These results con-firm that increasing ultrasound observation time improves the detection of fasciculations, particularly in muscles that are large or deep, without a significant loss of specificity in comparison with electromyography (Table 3).

**Table 3:** Comparison between electromyography and ultrasound across different observation times (10, 30, and 60 s), for detection of fasciculations.

	EMG		US	
		10 s	30 s	60 s
Muscle	(%)	(%)	(%)	(%)
Sternocleidomastoid	12	26	-	-
Tongue	11-55	68	63	85
Biceps brachii	61	-	88	83
First dorsal interosseous	58	-	78	82
Vastus lateralis/medialis	45-74	-	71	88
Tibialis anterior	47	-	82	91
Thoracic paravertebral	-	-	-	100
All muscles combined	43-49	50	59	80

EMG, electromyography; US, ultrasonography.

## DISCUSSION

This systematic review confirms that the diagnos-tic performance of ultrasound is superior to that of electromyography for detecting fasciculations in ALS, across all observation times and muscle groups. Previous studies have demonstrated the overall accuracy of ultra-sound^**(^28^,^29^)**^, and the present update underscores those findings through an expanded analysis including 10-, 30-, and 60-s protocols, with stratification by muscle.

It is important to note that the sensitivity of electro-myography also increases with longer examination times. Prolonged, careful sampling improves the likelihood of detecting fasciculations, particularly in muscles with low firing frequency, and that could partially reduce the performance gap between electromyography and ultrasonography in optimized protocols.^**(^30^)**^

At 10 s and 30 s, detection rates were comparable in general, yet ultrasound consistently identified a greater number of fasciculations within each muscle.^**(^31^,^32^)**^ The difference was particularly evident in muscles that are larger or deeper-such as the biceps brachii, vastus lateralis, and tibialis anterior-in which the sensitivity of electromyography tends to decline because of limited needle sampling area and muscle depth.^**(^33^)**^ In contrast, muscles such as the tongue and first dorsal interosseous, which are more superficial and easily relaxed, showed smaller differences between techniques.

Two complementary studies also evaluated 30-s observation protocols using distinct methodological ap-proaches. Botter et al.^**(^21^)**^ conducted a technical valida-tion study in healthy volunteers, demonstrating a strong correlation between surface electromyography signals and fasciculations visually detected on ultrasound. Bibbings et al.^**(^22^)**^ applied automated video processing through a motion-detection algorithm (“foreground detection”), comparing electromyographic findings with those of 30-s ultrasound sequences in ALS patients. Al-though neither study was included in the main clinical meta-analysis, both underscore the diagnostic accuracy of ultrasound and highlight its technological evolution for the identification of fasciculations.

In studies extending the ultrasound observation time to 60 s, detection rates increased substantially. Bokuda et al.^**(^14^)**^ and Rajula et al.^**(^15^)**^ demonstrated that longer dynamic evaluation improves the identification of fasciculations, particularly in muscles that are large or deep, such as the vastus lateralis, tibialis anterior, and paravertebral muscles. For proximal muscles such as the biceps brachii, which was assessed in both stud-ies, the authors found the performance of ultrasound to be equivalent or superior to that of electromyography, even under blinded conditions and with longer scan durations. These results confirm that extending the scan duration enhances the likelihood of visualizing spontaneous fasciculations that occur intermittently or at low frequency. When all observation times were pooled, ultrasound maintained higher detection rates than electromyography across all muscles, which con-firms the robustness and consistency of this finding.

The higher sensitivity of ultrasound in comparison with electromyography can be explained by its ability to monitor a broader muscle volume and detect con-tractions arising from multiple motor units, whereas intramuscular electromyography registers activity from a limited number of fibers surrounding the needle tip.^**(^34^)**^ In addition, ultrasound has the advantages of being noninvasive, painless, and widely available, while al-lowing simultaneous assessment of muscle atrophy and architecture.^**(^14^)**^ Furthermore, it has been reported that interobserver agreement for identifying fasciculations on ultrasound is excellent, underscoring the reproduc-ibility of the method.

The morphological characteristics of fasciculations may also provide diagnostic clues. Benign fasciculations tend to be stable, regular, and of lower amplitude, reflect-ing preserved motor-unit integrity. In ALS, fasciculations are typically unstable, variable in morphology, and ir-regular in frequency, often accompanied by fibrillation potentials and positive sharp waves on electromyography.^**(^35^-^37^)**^ Although ultrasound reliably displays the mechanical expression of fasciculations, it cannot differentiate between stable and unstable discharges, because that distinction depends on the characterization of motor-unit potential morphology and electrophysiological aspects.^**(^26^,^29^)**^ Therefore, although ultrasound is better at detecting fasciculations, electromyography is still essential for distinguishing benign fasciculation syndrome from pathological fasciculations associated with ALS.^**(^35^)**^

Electromyography continues to be the only technique capable of distinguishing between stable and unstable fas-ciculations through motor-unit potential analysis-a ma-jor limitation of ultrasound that can impede disease stag-ing and prognosis. Therefore, the two techniques should be regarded as complementary rather than competitive: electromyography provides detailed electrophysiologi-cal characterization, whereas ultrasound offers broader visualization and a dynamic context.

### Descriptive synthesis (unpooled analysis)

Across the two studies employing 60-s ultrasound

protocols^**(^14^,^15^)**^, the mean detection rates varied by muscle group, ranging from 80% to 95% for 60-s ul-trasound and from 65% to 80% for electromyography. The sensitivity gain with ultrasound was particularly pronounced in the vastus lateralis, tibialis anterior, and paravertebral muscles, confirming that a longer obser-vation time improves sensitivity, especially in deep or slow-contracting muscles.

### Study limitations

Our study has some limitations, most of which relate to the quality and heterogeneity of the primary studies included. The studies varied in muscle selec-tion, sample size, and observation protocols, which restricted the feasibility of pooled meta-analyses for certain muscle groups. Many investigations were con-ducted in single-center settings with relatively small ALS cohorts, and in several cases the operators were aware of the clinical diagnosis-factors that might have intro-duced selection or observer bias. In addition, because the reference standard relied on technical rather than clinical comparisons, the results reflect relative detection performance rather than absolute diagnostic accuracy. Reporting of interobserver agreement was inconsistent, and the absence of standardized protocols for image acquisition and interpretation continues to be a barrier to broader clinical applicability. Despite these study-level constraints, the present review adhered strictly to the principles established in the Cochrane Handbook for Diagnostic Test Accuracy Reviews, which ensured a high standard of methodological rigor. Independent dual data extraction and structured quality appraisal were employed to minimize bias and enhance transparency. This robust, systematic approach supports the reliability of our conclusions and provides the most comprehen-sive synthesis to date on detection of fasciculations by ultrasound and electromyography in ALS.

### Clinical implications and future perspectives

Although the aforementioned limitations must be considered, our findings underscore the clinical utility of muscle ultrasound as a complementary diagnostic tool and, in some cases, a tool for earlier diagnosis in ALS. Ultrasound provides a noninvasive approach to screening for fasciculation and is particularly valuable in muscles that are large or deep, in which electromyography has limited sensitivity. Integrating real-time ultrasound into neurophysiological assessment may enhance diagnostic accuracy, reduce the number of electromyography inser-tions, and improve the patient experience.

Future multicenter studies with standardized muscle selection, observation times, and interpretation criteria are needed in order to establish consensus protocols. Emerging technologies-such as automated motion detection and artificial intelligence-based analysis-may further improve reproducibility and allow objective quan-tification of fasciculation frequency and morphology over time. Such advances could enable ultrasound not only to complement electromyography but also to serve as a longitudinal biomarker of disease progression and treatment response in ALS.

## CONCLUSION

In several muscle groups and across observation protocols, ultrasound demonstrated higher rates of de-tection of fasciculations in ALS than did electromyog-raphy. That advantage was more evident in muscles that are large or deep-such as the vastus lateralis, tibialis anterior, and paravertebral muscles-particularly when longer observation times were applied, suggesting that extended scanning increases sensitivity without com-promising specificity.

By enabling real-time visualization of superficial and deep musculature, ultrasound allows broader sampling of motor units and facilitates the identification of spontane-ous muscle activity that may be missed by intramuscular electromyography. However, electromyography continues to be essential for characterizing motor-unit potential morphology and for distinguishing between stable and unstable fasciculations.

Taken together, our findings support the role of ul-trasound as a complementary, noninvasive, reproducible tool that can enhance the assessment of LMN dysfunc-tion, improve patient comfort, and potentially reduce the need for extensive needle sampling. The combined use of ultrasound and electromyography may therefore provide a more comprehensive evaluation strategy. Multicenter studies with standardized acquisition protocols and the incorporation of automated image-analysis techniques are warranted to further define the role of ultrasound as a quantitative biomarker for monitoring disease pro-gression and treatment response in ALS.
